# Disuse Impairs the Mechanical Competence of Bone by Regulating the Characterizations of Mineralized Collagen Fibrils in Cortical Bone

**DOI:** 10.3389/fphys.2019.00775

**Published:** 2019-06-21

**Authors:** Peng-Fei Yang, Xiao-Tong Nie, Zhe Wang, Luban Hamdy Hameed Al-Qudsy, Li Ren, Hui-Yun Xu, Joern Rittweger, Peng Shang

**Affiliations:** ^1^Key Laboratory for Space Bioscience and Biotechnology, School of Life Sciences, Institute of Special Environmental Biophysics, Northwestern Polytechnical University, Xi’an, China; ^2^Research & Development Institute, Northwestern Polytechnical University, Shenzhen, China; ^3^Yangtze River Delta Research Institute, Northwestern Polytechnical University, Taicang, China; ^4^Division of Muscle & Bone Metabolism, Institute of Aerospace Medicine, German Aerospace Center, Cologne, Germany; ^5^Department of Pediatrics and Adolescent Medicine, University of Cologne, Cologne, Germany; ^6^Key Laboratory for Space Bioscience and Biotechnology, Institute of Special Environmental Biophysics, Northwestern Polytechnical University, Xi’an, China

**Keywords:** collagen fibril, bone, disuse, mechanical properties, atomic force microscopy

## Abstract

Bones are made of complex material comprising organic components and mineral hydroxyapatite, both of which formulate the unique hierarchical structure of bone and its mechanical properties. Bones are capable of optimizing their structure and mechanical properties according to the mechanical environment. Mineral loss is a well-known consequence of skeleton disuse. By contrast, the response of the non-mineral phase of bone, i.e., the collagen network, during disuse remain largely unknown. In this study, a tail-suspension mice model was used to induce bone loss. Atomic force microscopy-based imaging and indentation approaches were adopted to investigate the influence of disuse on the morphology and *in situ* mechanical behavior of the collagen fibrils, under both non-loaded and load-bearing conditions, in the cortical tibia of mice. The results indicate that disuse induced by hindlimb unloading did not alter the orientation and D-periodic spacing of the collagen fibril, but results in decreased collagen crosslinking which correlates with decreased elasticity and increased susceptibility to mechanical damage. More concretely, the collagen fibrils in the disused tibia were misaligned under mechanical loading. It therefore indicates that the disordered arrangement of the mineralized collagen fibrils is one of the characteristics of the weakened bone during elastic deformation. These findings reveals the unique adaptation regimes of the collagen fibrils in the cortical bone to disuse, as well as the deformation mechanisms of bone in the relevant pathological process at different scales.

## Introduction

Bone is a hierarchical-structured material that comprises organic components and mineral HAP, and the unique structure of bone governs its mechanical properties. One of the most remarkable characteristics of bone is that it optimizes its structure and mechanical properties according to the surrounding mechanical environment (Skerry, 2008). Bone mineral loss is one of the well-known consequences of musculoskeletal disuse. Generally, mineral loss is accompanied by mechanical deterioration of the bone structure during disuse. The mechanical properties of the cortical bone play a decisive role in bone fracture resistance. Therefore, the mineral phase of bone has long been considered as an indicator of bone quality and fracture risk.

Collagen is the most abundant protein in the bone matrix, and forms a three-dimensional (3D) template of the collagen network for bone apatite mineralization. The mineral HAP is deposited in the gap regions of the adjacent collagen molecules or on the surface of the collagen fibrils. This reinforced hierarchical structure on the bases of the MCFs plays a critical role in the mechanical competence of bone. Recent evidence indicated that the organic phase in the bone matrix, particularly Type I collagen, is also vital for the mechanical behavior of bone (Garnero, 2015). Long-term running training significantly reduced BMD of dogs and reorganized bone collagen fibrils in a more parallel manner (Puustjarvi et al., 1999). Surprisingly, despite the decrease in the BMD, the well-organized collagen network is still able to maintain the strength properties of bone (Puustjarvi et al., 1999). In contrast, significant changes in collagen morphology can be observed in murine models of osteoporosis (Silva et al., 2006) and of bone cells dysfunction (Hammond et al., 2016). Furthermore, studies also show that the maturity of the collagen cross-link correlates with the fracture toughness of bone (McNerny et al., 2015).

Bone mineral density and BMC, as well as bone microstructure, are the most common indicators that represent the status of bone loss. It is well known that tail-suspension-induced disuse could induce bone mineral loss and the weakness of bone mechanical properties (Morey-Holton and Globus, 1998, 2002). During disuse, bone resorption primarily occurs on its endosteal surface due to the activities of osteoclasts. By contrast, although cortical compartment dominates the overall mechanical integrity of bone, cortical bone loss during disuse has attracted less attention. Extensive studies in rodents showed that tail suspension induced both cortical and trabecular bone loss at the hind limbs (Wronski and Moreyholton, 1987; Meakin et al., 2014), although only in some studies, few changes were observed in the cortical bone (Bloomfield et al., 2002). For instance, endcortical bone loss or expansion was observed in the female B6 mice subjected to sciatic neurotomy (Kodama et al., 1999). In astronauts, mineral loss and redistribution of bone minerals in the cortical bone were also observed (Lang et al., 2004). Increased intracortical porosity, decreased bone geometrical properties and decreased bone mineralization were also observed on the cortical bone during disuse (Gross and Rubin, 1995; Kaneps et al., 1997; Li et al., 2005). More importantly, the mechanical weakness of bone often accompanies with mineral loss.

Cortical bone has a typical hierarchical structure, and collagen is one of the basic components of such a highly hierarchical structure. The morphology and organization of the collagen fibrils were shown to be closely related to the mechanical integrity of the bone (Garnero, 2015). With a murine model of senile osteoporosis, Silva et al. (2006) showed that weakness of the bone matrix is attributed to the alterations in collagen content and poor collagen fiber organization. However, the response of the collagen network in cortical bone to disuse, as well as its influence on the macroscopic mechanical properties of bone from the perspective of material components of bone, remain largely unknown. The collagen fibers are constructed with both the MCFs and the cross-links among the fibrils. It can therefore be logically deduced that both the mechanical properties of the MCFs and the cross-links between the fibrils contribute to the overall mechanical behavior of the collagen network and bone matrix. Nevertheless, the *in situ* mechanical properties of the MCFs in bone matrix have been technically difficult to assess. The ways in which the mechanical properties of the MCFs in bone respond to disuse and the potential contribution of *in situ* mechanical properties of the MCFs to the mechanical competence of bone at different scales have been largely overlooked.

Hence, in the present study, we hypothesized that the morphology and *in situ* mechanical properties of the MCFs were changed during disuse, and that they were closely related to the mechanical properties of bone across different scales. To test this hypothesis, a tail-suspension murine model was used to induce bone loss of the lower limb. After partial demineralization, the morphology of the MCFs in the partially demineralized bone surface was scanned using AFM. The nano-indentation approach based on AFM was adopted to assess the *in situ* mechanical properties of the single MCF. Nano-indentation was used to assess the micro-scale mechanical properties of the partially demineralized bone surface. Bone deformation regimes of both the normal and the disused bone at the nanoscale level under mechanical loading were analyzed and discussed.

## Materials and Methods

### Experimental Design

Twenty-four BALB/c male mice were purchased from The Lab Animal Center of the Fourth Military Medical University (Xi’an, Shaanxi, China). The animals were allowed to acclimate for 2 weeks prior to the study until they were 10 weeks of age. All mice were given standard rodent chow and water *ad libitium* with individually housing at 25° C and in a common light/dark cycle. The animal experiments have the “Laboratory Animal Management Regulations” promulgated by Decree No. 2 of the State Science and Technology Commission of China. The entire experiment was approved by the animal ethics and welfare committee of the Northwestern Polytechnical University.

The mice were randomly assigned to two groups: age-matched control group, and tail suspension group (12 mice for each group). Hindlimb disuse was induced by tail suspension. Sample size of the animals in each group was determined by calculating the statistical power to establish the bone loss model (Shirazi-Fard et al., 2013). The duration of the experimental protocols was 4 weeks. Bilateral tibia and femur of mice was harvested on day 28 for the control and tail suspension groups. The bone samples were cleaned to remove the adjacent soft tissue, wrapped in gauze soaked with PBS, placed in a sealed tube, and stored at −20°C for future measurements. The body weight was recorded weekly throughout the experiment. The overall BMD, BMC, microstructure, and macro-scale mechanical properties of the tibia were quantified with X-ray based techniques and three-point bending test prior to the other micro- and nanoscale assessments. Considering that the changes of these characteristics of the tibia to disuse in murine tail-suspension models have been extensively investigated in the past and the macro-scale alterations of bone were rather apparent, therefore, the corresponding results were provided in the Supplementary Materials only.

### Axial Tibia Loading Model Under AFM

In order to assess the changes of the MCFs in tibia from different groups to mechanical loading, a murine tibia loading device, which can be used for *in situ* AFM scanning, was custom-built, as described previously (Yang et al., 2018). A piezoelectric actuator (PK2FVF1, Thorlabs, NJ, United States) was used to generate the precise mechanical force on the tibia. Then, the mechanical force was monitored using a force sensor (L6D21, Zhonghang Electronic Measuring Instruments Co., Ltd., Hanzhong, China) as the feedback. The tibia was preloaded with 0.5 N to avoid loosening in the loading device. The preloading condition was referred to as the pre-load condition in the following text. Our previous study showed that the most dramatic changes of the MCFs are induced by the mechanical loading of 6 N (Yang et al., 2018). Therefore, pre-load and 6 N were chosen to load the tibia at each session of the AFM scanning. The stress relaxation of the bone was monitored in the axial loading model. During the AFM scanning, the variation of the loading amplitude was less than 0.5 N, which was lower than the pre-loading amplitude, and was considered to be negligible. Moreover, five minutes rest time was given between the adjacent scans to let the bone recover and avoid the potential influence of previous loading on bone morphology at the level of the collagen fibrils. Previous finite-element analyses indicated that the scanning sites were under tension with the average strain ranging from 1000 to 1500 με (Yang et al., 2018). Digital Image Correlation approach (Guo et al., 2017) was adopted to quantify the surface strain of tibia from both the control and tail suspension groups. Results indicated that 6 N results 1960 ± 240 με bone strain for control group and 2498 ± 310 με bone strain for the tail suspension group (*p* = 0.08, Supplementary Materials). The 538 με difference in average between the control and tail suspension groups was not the main consideration in the present study, because the scope of the present study is not on the collagen response under the equal amount of bone surface strain, but on the load-bearing capability and mechanical behavior of bone. The present study focused on the relative, rather than absolute, alterations of normal and disused bone to mechanical loading, and the alterations covered multiple scales, therefore the loading amplitude acting on bone was not normalized to specific-scale formation, i.e., local tissue strain.

### AFM Image Scanning

While the tibia was fixed on the axial loading device, a relatively flat surface in the antero-medial aspect of the tibia was chosen for the AFM scan (six tibias from six different mice). The sample preparation, demineralization and AFM scanning procedure were described in our previous publication (Yang et al., 2018). In detail, the cortical bone surface of the chosen sites were polished using 7000 grit sand paper, 3-μm diamond suspension, and 0.5-μm diamond suspension in sequence. Each sample was sonicated in ultrapure water for 5 min to remove the polishing residues and debris. In order to remove the extra fibrillar mineral and expose the MCFs, bone samples were then treated with 0.5-M EDTA at a pH of 8.0 for 15 min at 30°C, followed by rinsing in ultrapure water for 5 min. The demineralization procedure was then repeated three times. EDTA treatment is a common way to remove the superficial mineral of the mineralized tissue while remaining the native collagen structure and visible. This demineralization method described above has been validated in many previous studies (Balooch et al., 2008; Wallace et al., 2010, 2011).

Bone samples (six tibias from six different mice) were scanned in air under pre-load and 6 N conditions using a PicoPlus 5500 AFM (Agilent, MI, United States). Images were acquired in tapping mode using a silicon scanning probe (PPP-NCL-20, resonance frequency: 146–236 kHz, force constant: 21–98 N/m, Nanosensors, United States). Images were taken from the field-of-view of 10 × 10 μm and further 5 × 5 μm at a matrix size of 512 × 512 pixels from three sites on each bone sample, with a distance of approximately 1 mm along the long axis. The scanning speed was set at 1.2 line/s (Wallace et al., 2011; Hammond et al., 2016). The entire procedure was completed on the dehydrated bone surface.

### Image Analysis

Images with the size of 2 × 2 μm were cropped from each AFM scanned image for D-periodic spacing and orientation analysis of the MCFs. Depends on the image quality, images of three bones chosen from six tibias were used for D-periodic spacing, fibril organization and radial elastic modulus calculations. The detailed procedure of AFM image analyses was also described below (Yang et al., 2018).

#### D-Periodic Spacing of the MCFs

Ten MCFs from each AFM amplitude image in different bone sites were labeled manually along the long axis of the fibril with a commercial software (PicoView, Agilent, MI, United States). The length of the labeled fibril and number of D-periodic feathers involved were different from image to image. Therefore, the total number of data for statistics would be different. The amplitude data of the MCFs were analyzed with custom-written Matlab routine (Mathworks, Inc., MA, United States). The D-periodic spacing of the MCFs was determined by calculating the distance between the adjacent peaks of the deviation amplitude curve.

#### Orientation of the MCFs

Twenty MCFs from each image (three images/tibia, three tibias/group). (180 MCFs in total were chosen for statistics from each group) were chosen and manually labeled along the long axis of the fibrils using a commercially available software (Gwyddion, Version 2.22, Czech Metrology Institute, Czechia) in 2 × 2 μm AFM images, as demonstrated in our previous paper (Yang et al., 2018). The acute angle between the labeled line of the MCFs and the horizontal axis of the image was calculated. The obtained orientation angles were normalized by subtracting the most concentrative distributed angle among these 20 chosen orientation angles.

### *In situ* Radial Elastic Modulus of MCFs

*In situ* mechanical properties of the exposed MCFs after partial demineralization were measured using the AFM-based nano-indentation approach. Immediately following the morphological scanning of the bone surface, AFM was switched to the contact mode for nanoindentation tests of a single MCF. The same AFM probe (PPP-NCL-20, Nanosensors, United States, inverted pyramidal tip with a 20° side angle) from image scanning was used to assess the radial mechanical properties of the single MCF. The longitudinal elastic modulus of the MCFs would be the ideal indicator of the mechanical properties of the MCFs, because it directly relates to the overall mechanical properties of bone. However, to date, it is still technical challenging to assess the *in situ* longitudinal elastic modulus of the MCFs. The present study therefore adopted the radial elastic modulus instead. In order to minimize of the potential influence of the mineralization on the elastic modulus measurements, the present study used the same demineralization approach to process both the control and tail suspension bone. Twenty self-chosen MCFs from each image were chosen under the navigation of each morphology image of the collagen fibrils to perform nanoindentation tests. Three images were collected from different AFM scanning sites in each tibia. Therefore, 60 MCFs were measured in each tibia. In total, radial elastic modulus of 180 MCFs were assessed from each group. The spring constant of the cantilever was obtained using the “Thermal K” approach (Agilent Technologies, MI, United States). The distance–deflection relationship was calibrated by placing the probe against a mica plate prior to each test. The indentations were performed at low piezo-displacement speeds (2.5 μm/s), and for the last 4.8 s (20,000 sampling points) to minimize the viscosity effects. The force-displacement curve during the indentation was recorded and converted into a force-indentation curve. The *in situ* transverse stiffness (Elastic modulus, *E*) of the MCFs was determined using our previously described approach (Yang et al., 2018). In detail, the contact point of the probe to the MCFs was identified with a custom-written MATLAB algorithm from the force-indentation curve with a noise-determination method (Crick and Yin, 2007). The Hertzian contact model (Lin et al., 2007; Carl and Schillers, 2008) was adopted to fit the selected force-indentation curve with a custom-written MATLAB routine. The *in situ* transverse stiffness of the MCFs was determined using the following equation:

(1) $$F=\frac{E}{1-v^{2}}\,\frac{t\,a\,n\,\alpha }{\sqrt{2}}\,\delta^{2}$$

This equation could also be transformed into the alternative expressions of:

(2) $$f_{\mathrm{cone}}=\frac{2}{\pi }\,\frac{E}{1-v^{2}}\,t\,a\,n\,\alpha \,\delta^{2}$$

(3) $$E=\frac{2}{\pi }\,{\left(\frac{\Delta \,{\left(f_{\mathrm{cone}}\right)}^{1\,/\,2}}{\Delta \,\delta }\right)}^{2}\,\frac{1-v^{2}}{t\,a\,n\,\alpha }=\frac{\pi }{2}\,s\,l\,o\,p\,e^{2}\,\frac{1-v^{2}}{t\,a\,n\,\alpha }$$

where *F* is the indentation force, *E* is the elastic modulus of the collagen fibril, α is the side angle of the pyramidal indenter, δ is indentation distance, and *v* is the Poisson’s ratio. In the present study, *v* was assumed to be equal to 0.3. The variable *f_cone_* represented the pyramidal indenter.

### Raman Spectrometry

Six tibia samples from six different mice in each group were dehydrated with rising series of ethanolic solutions and embedded in PMMA. 50-μm-thick sections were cut with a microtome (Leica, Germany). The sections were fixed on a microscope slide. Raman measurements were conducted with a Raman spectrometer (Invia RM2000, Renishaw, London, United Kingdom) using a laser with a wavelength of 785 nm, laser power of 50%, extend scan. The wave-number range of collected Raman scattered photons was 1800–800 cm^–1^ and baseline corrected using a polynomial fitting-based method. In order to match the locations of the collagen fibrils investigated in the present study, the measurement sites were chosen at the intracortical compartment of the antero-medial aspect of the tibia. A total of 36 images (one images/location, three locations/specimen, six specimens from the control group, and six specimens from the tail suspension group) were obtained to investigate the collagen cross linking of Pyr (mature) and DHLNL (immature). The peaks at 1660 and 1690 were located at the curve with custom-written Matlab routine (Mathworks, Inc., MA, United States) to indicate the cross linking of Pyr and DHLNL. Likewise, the primary phosphate band (approximately 959 cm^–1^) and the amide I band (1616–1720 cm^–1^) were identified to compute the mineral-to-matrix ratio, which indicates the amount of mineralization.

### Nanoindentation

At the micro-scale level, the *E* and hardness values of the same demineralized bone surface for AFM assessment were measured by nanoindentation (Hysitron Triboindenter TI-950, Minneapolis, Minnesota), as described previously (Grover et al., 2016). Indentation was conducted at three different sites on each tibia. Three tibias from three different mice were tested for each group. In total, nine indentations were conducted for the bone samples from each group. The micro-scale level covered more than single collagen fibril, thereby evaluating the mechanical properties of the mineralized collagen fibril network. More specifically, a Berkovich tip was used for all of the indentations. Indents were made on the exposed bone surface in the antero-medial region of the bone to maintain the measurement consistency among bone samples. The system was calibrated using indentation analysis on fused quartz prior to the measurements. The drift rates of the system were measured prior to the indentation tests using standard indentation testing procedures. The indentation consisted of a 10-s loading period at a constant loading rate of 100 μN/s. A constant load segment at the peak load of 1000 μN followed this for 30 s. In the end, the tip was retracted in the unloading segment for another 10 s at a constant unloading rate of 100 μN/s. The total indentation time was 50 s. The elastic response was calculated from the 20–95% portion of the unloading curve. The *E*-value was calculated assuming an elastic response during unloading, and using the Oliver Pharr method (Hoc et al., 2006).

### Statistical Methods

Data were expressed as mean ± SD. Statistical analyses were conducted with SPSS (Version 23, SPSS Inc.). In order to investigate the influence of the mechanical environment on the collagen fibril morphology, D-periodic spacing values, orientational angles, *in situ* elastic modulus of the MCFs, tissue-level mechanical properties, and the collagen crosslink of the individual bone from the same group were averaged. The inter-group difference was assessed using the unpaired Student’s *t*-test or one-way ANOVA test with Holm-Sidak’s multiple comparisons. The distribution of the MCFs was represented with histograms and the CDF of each group (Wallace et al., 2011). Statistical analyses on the difference between group distributions were performed with K–S tests. The statistical significance was accepted while the *p*-Value was 0.05 or less.

## Results

### Morphology of MCFs

**FIGURE 1:**
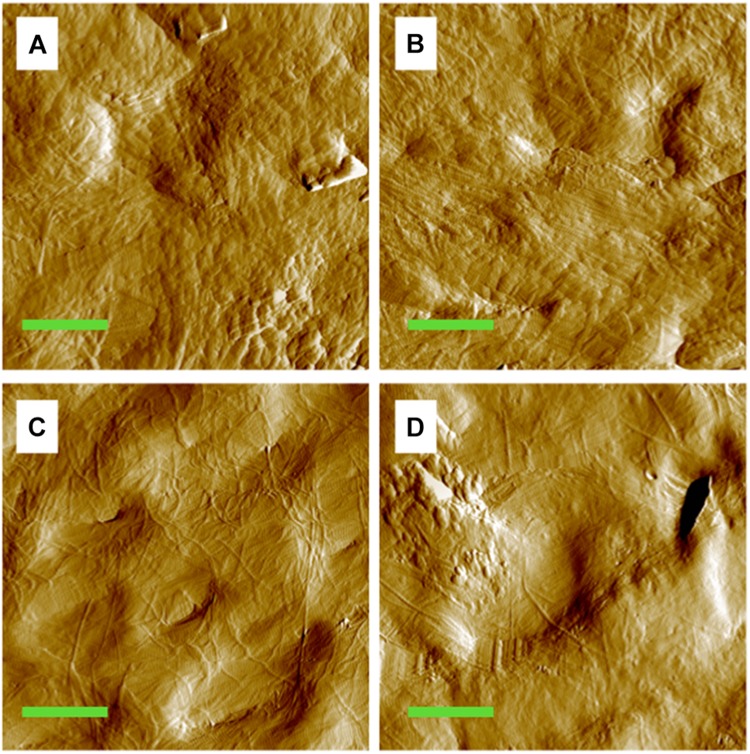
Amplitude image of the MCFs in the cortical bone of the mouse tibia. **(A)** Pre-loaded tibia from the control group. **(B)** Pre-loaded tibia from the tail suspension group. **(C)** Tibia from the control group under 6 N. **(D)** Tibia from the tail suspension group under 6 N. Green bar: 2 μm.

The MCFs in both the control (Figures 1A,C) and disused cortical tibia (Figures 1B,D) can be well discerned under different loading conditions. From inspection of the images, it seems that the morphology of the exposed collagen network is smoother and displays less texture under mechanical loading of 6 N (Figures 1C,D) comparing to the pre-load groups (Figures 1A,B). However, the visual quality of the AFM amplitude image may highly depended on the adopted threshold. In order to quantify the difference of the images, the D-periodic spacing and orientation of the MCFs were employed to identify the morphological alterations of the MCFs.

### D-Periodic Spacing of Control and Disused Bone

**FIGURE 2:**
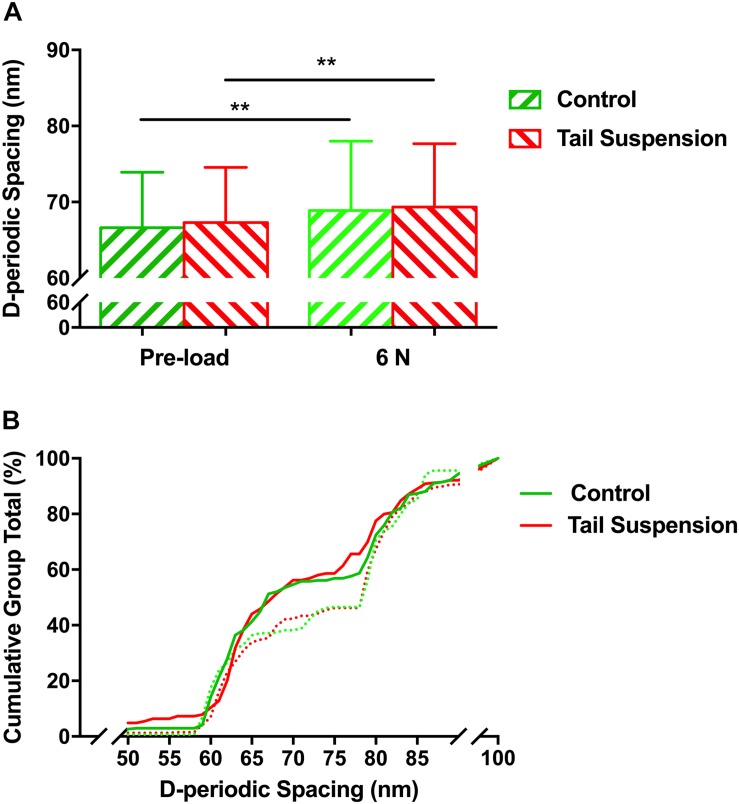
Histogram of mean values **(A)**, and the cumulative density function **(B)** of D-periodic spacing of the MCFs. There was no difference in the mean value of the D-periodic spacing of the MCFs in the tibia between the control and tail suspension group at a loading of either pre-load or 6 N. Kolmogorov–Smirnov statistics indicated significant differences in the distributions of the D-periodic spacing values between the loading of pre-load (solid line) and 6 N (dashed line) (*p* < 0.001). Data was generated from three bone samples of three different mice in each group. ^∗∗^*p* < 0.01.

The overall mean value of the D-periodic spacing of the MCFs was 67 ± 7.3 nm in the control group (*n* = 246) and 67 ± 7.2 nm in the tail suspension group (*n* = 287). For a mechanical loading of 6 N, the D-periodic spacing of the MCFs was increased to 69 ± 9.1 nm for the control group (*p* = 0.0077, *n* = 268) and 68 ± 7.9 nm for the tail suspension group (*p* = 0.0047, *n* = 284; Figure 2A). There was no difference in the D-periodic spacing of the MCFs among groups at the single loading condition of either pre-load (*p* = 0.31) or 6 N (*p* = 0.98). The population distribution of D-periodic spacing of the MCFs at 6 N (Figure 2B, dashed lines) has similar trend compared to the value at pre-load (*p* < 0.001; Figure 2B, solid lines). However, the population distribution value of D-periodic spacing ranging from 65 nm to 80 nm was very different.

### Orientation Angles

**FIGURE 3:**
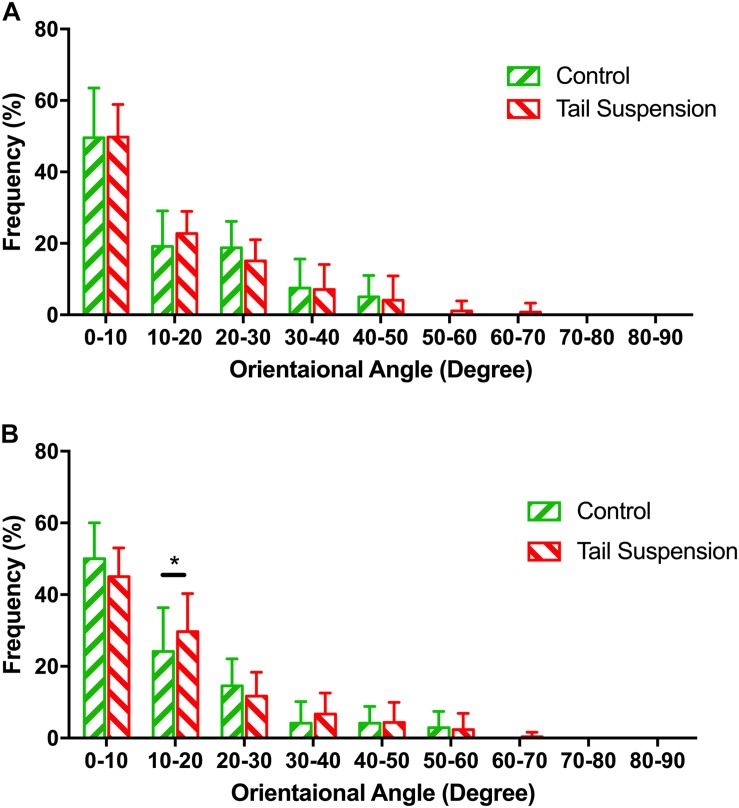
Distribution of the normalized orientation angles of MCFs in different groups at pre-load condition **(A)** and 6 N **(B)**. The figure shows the frequencies at which the orientation angles of the MCFs appear in the acquired AFM images were shown. Kolmogorov–Smirnov tests indicated significant differences among different groups under 6 N. Data was generated from three bone samples of three different mice in each group. ^*^p < 0.05.

No difference was found in the orientation distribution of the MCFs among groups at the loading of pre-load (*p* > 0.990), thereby indicating that tail-suspension-induced disuse did not affect the orientation angle of the MCFs from the perspective of its original morphology (Figure 3A). In contrast, at 6 N, the orientation angles of the tail suspension group were more distributed than the control group in the range of 10–20° (*p* = 0.027).

### *In situ* Radial Elastic Modulus

**FIGURE 4:**
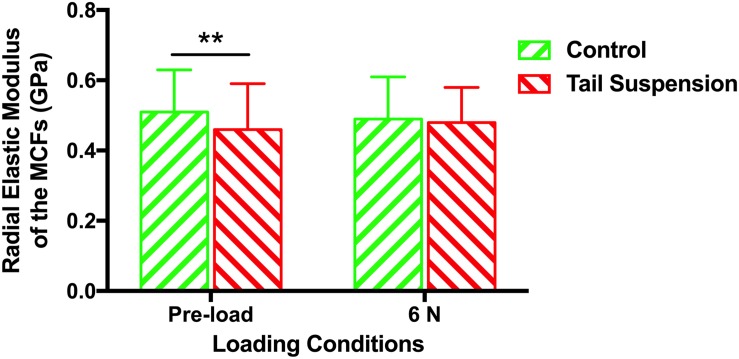
Histogram of the *in situ* radial elastic modulus of the MCFs in cortical tibia of different groups under loading conditions of pre-load and 6 N. Data was generated from three bone samples of three different mice in each group. ^∗∗^*p* < 0.01.

The radial elastic moduli of the MCFs were 0.51 ± 0.12 GPa and 0.46 ± 0.13 GPa for the control and tail suspension groups (*n* = 180 for each group), respectively. The radial elastic modulus of the MCFs of the tail suspension group was 10% lower than the control group (*p* = 0.0095). With a mechanical loading of 6 N, the radial elastic moduli of the MCFs were 0.49 ± 0.12 GPa and 0.48 ± 0.10 GPa for the control and tail suspension group, and it remained at the same level between two groups (*p* = 0.19; Figure 4).

### Mechanical Properties of Tibia at Tissue Level

**FIGURE 5:**
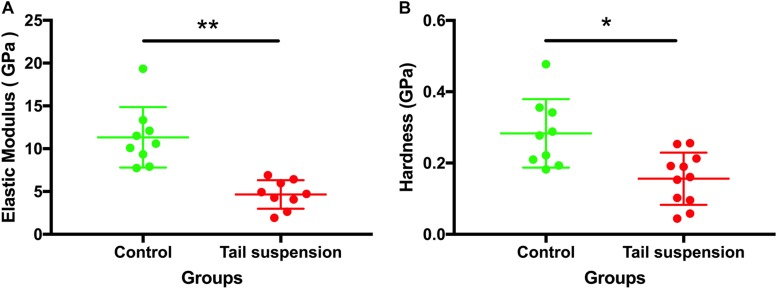
Tissue-level mechanical properties of the tibia. Elastic modulus and hardness of the antero-medial cortical tibia were derived and represented from the nanoindentation assessment. Data was generated from three bone samples of three different mice in each group. ^*^p < 0.05 and ^∗∗^*p* < 0.01.

At the tissue level, the elastic modulus (*E*) of the cortical tibia in the control group was larger than those of tail suspension group (11 ± 5.5 GPa for the control group vs. 4.7 ± 1.7 GPa for the tail suspension group, *p* = 0.0066, *n* = 9; Figure 5A). Likewise, the hardness of the control group was also larger than those of the tail suspension group (0.28 ± 0.12 GPa and 0.16 ± 0.073 GPa, *p* = 0.040; Figure 5B).

### Raman Spectrometry Results

The ratios of 1660 and 1690 underlying the amide I band are particularly indicative of the cross linking of Pyr and DHLNL. Statistical evaluation suggested that the collagen crosslink ratio in the tail suspension group was 38.9% lower than the control group (*p* = 0.011; Table 1). The mineral-to-matrix ratio of bone were 0.06 ± 0.01 for the control group and 0.09 ± 0.03 for the tail suspension group, indicating that small amount of minerals remained on the bone surface.

**TABLE 1:** Statistical result of the collagen crosslink ratioamong different groups (*n* = 6).

**Group**	**Control**	**Tail suspension**
Collagen crosslink ratio	1.80 ± 1.30	1.10 ± 0.36^*^

Data was generated from six bone samples of six different mice in each group. ^*^p < 0.05.

## Discussion

The response of murine cortical bone to tail-suspension-induced disuse at multiple scales was demonstrated in the present study. Our results show that tail-suspension-induced disuse impairs the tissue-scale mechanical properties of the tibia (Figure 5), very likely by reducing the *in situ* mechanical properties of the mineralized collagen fibrils in the murine cortical tibia instead of changing the morphology and orientation of the fibrils. Disuse results in decreased collagen crosslinking which correlates with decreased elasticity and increased susceptibility to mechanical damage. In detail, it is shown that the orientation of the mineralized collagen fibrils in the disused bone responds differently compared with the response of the healthy bone under loading (Figure 3B). This therefore indicates that the disordered arrangement of the mineralized collagen fibrils is one of the characteristics of the weakened bone during elastic deformation. Of note, different tissue strain between the healthy bone and the disused bone may contribute to the different response of the mineralized collagen fibrils under the equivalent mechanical loading. These findings revealed the process and mechanisms of how disuse impairs the mechanical competence of bone at different scales.

The present study demonstrated that 28-day tail suspension disuse indeed had the greatest bone mineral loss, impaired the microstructure and the macro-scale mechanical properties of murine tibia (Supplementary Materials). In particular, the cortical area and cortical BMC, as well as the major mechanical parameters of tibia shaft, decreased after 28-day tail suspension (Supplementary Materials). The macro-scale strength of the long bone is mainly determined by its outer cortical shell. At the nanoscale, disuse appears to directly influence the mechanical properties of the single MCF, but interestingly, at the nanoscale, neither the D-periodic spacing nor the orientation of the MCFs in the cortical bone was directly affected by tail suspension, while there is no mechanical load acting on bone (Figures 2, 3). One of the possible explanations is that the mineral loss in the cortical compartment remains insufficient to alter the morphology of the collagen network, although evidence suggested that mineral loss could alter the morphology of the collagen fibrils in bone (Puustjarvi et al., 1999). It is apparent that the cortical bone is less accessible to be resorbed than trabecular bone during disuse, although evidence indicates that intracortical bone remodeling is responsible for the largest bone loss in osteoporosis (Zebaze et al., 2010).

The present study revealed that the radial elastic modulus of the MCFs was decreased by tail-suspension-induced disuse, which is congruent with previous findings at the nanoscale level (Balooch et al., 2008). The demineralization process was capable of dramatically reducing the elastic modulus of the collagen fibrils (Balooch et al., 2008). On the contrary, the elastic modulus of the MCFs also increased with the extent of mineralization (Chatzipanagis et al., 2016). Not only at the nanoscale level, but the impairment of disuse on the mechanical properties of bone was also observed at the microscale (Figure 5) using the indentation approach, and at the macroscopic level (Supplementary Materials) using the three-point bending test. Typically, the indentation tests should be performed in the longitudinal direction of the fibrils. However, to date, assessing the *in situ* longitudinal elastic modulus of single collagen fibril is still technical challenging. Moreover, Casanova et al. (2017) also suggested that the transverse elastic modulus of the collagen network at the microscale level also has significant impact on the mechanical behavior of bone (R Soc Open Sci). In order to ensure comparability between the AFM nano-indentation tests and the tissue-level nano-indentation tests, we performed the transvers measurements at both the nanoscale (single collagen fibrils) and microscale level (tissue-level nano-indentation). Previous study also showed that the morphology alterations of collagen in bone at the nanoscale result in compositional and mechanical changes at the microscale (Hammond et al., 2014). However, the present study suggested that murine tibia respond to disuse differently at nanoscale, microscale and macroscopic levels. It indicated that both the mineral phase and collagen contribute to the larger scale mechanical behavior of bone. However, the exact mechanism behind and the mineral-collagen interaction during loading bearing condition deserve further investigation.

Bone has to resist the external load during daily life. The morphology and mechanical properties assessed under the pre-load status of bone may not be able to represent the capability of bone to resist mechanical loading. Moreover, the morphology and mechanical behavior of bone may also be different at the nanoscale while bone is under mechanical loading. The present study further assessed how the alterations of bone at the nanoscale level influence the capability of bone to resist mechanical loading. Thus, an axial tibia loading model was adopted in the present study to determine the response of the murine tibia to mechanical loading at the nanoscale. Details of the loading protocol have been described in our previous publication (Yang et al., 2018). Given that our previous study has shown the deformation regimes of collagen fibril stretching and sliding under the mechanical loading of 6 N (Yang et al., 2018), 6 N was therefore chosen to load the murine tibia from different groups.

Under 6 N condition, the MCFs in antero-medial cortical tibia from all groups were stretched. The *in situ* radial elastic modulus of the single MCF was equivalent in all groups under the mechanical loading of 6 N. Disuse dramatically reduced the elastic modulus of the MCFs. Under mechanical loading, the elastic modulus of the MCFs from different groups was brought back to the same level. Based on the orientation and radial elastic modulus data of the MCFs, it appears that the disuse impaired MCFs were stretched more than normal MCFs, and became “stiffer” under equal amounts of mechanical loading. In this sense, the disused bone had a larger tissue deformation than the normal bone under the same amount of mechanical loading, which has been demonstrated in Supplementary Materials. However, high tissue strain does not necessarily induce more fibril stretching, but may involve different deformation regimes, e.g., fibril stretching and fibril sliding (Yang et al., 2018). Therefore, more fibril stretching of the disused bone under mechanical loading was one of the present findings. The changes of the elastic modulus alongside with fibril stretching was another finding in the present study.

The orientation pattern of the MCFs is a crucial parameter that is related to the local mechanical properties of bone (Martin and Ishida, 1989; Schrof et al., 2016). Fibril orientation is not only a parameter that represents the nanoscale structure of the bone, but it also plays a significant role in the mechanical properties of single osteon (Granke et al., 2013). The present study revealed that the orientation of the MCFs was not affected by disuse. However, under mechanical loading, the MCFs of the disused bone became more dispersed than the normal ones (Figure 3). This therefore indicated that the removal of bone minerals may exaggerate the disorganization of the MCFs, particularly when the bone bears the external load. The misalignment of the MCFs in disused bone during deformation may indicate that disuse indeed weakens the load-bearing capability of bone at the nanoscale level. Higher tissue strain could be one of the reasons of the dispersed MCFs. However, theoretically, higher strain normally lead the MCFs more parallel prior to the rupture of the MCFs. Results of our previous study indicated that bone tissue undergoes different deformation regimes with variations in the amplitude of mechanical loading (Depalle et al., 2015, 2016; Yang et al., 2018). According to our previous study, the MCFs start to slide relative to each other at 6 N (Yang et al., 2018). It appears that the equivalent amount of mechanical loading inhibited the collagen-sliding process of the disused bone, comparing to that of the control bone, by altering the organization of the MCFs. The load of 6 N was the only loading condition that was tested in the present study. Previous data indicated that the murine tibia continues to experience elastic deformation under 6 N (Yang et al., 2018). It remains to be determined whether the MCFs in the disused bone still respond differently to the normal bone under the other loading conditions. Likewise, how different tissue strain influence the organization of the MCFs in the disused bone should be further investigated.

The collagen cross-linking is closely related to the mechanical properties of bone (Berteau et al., 2015; Depalle et al., 2015; McNerny et al., 2015). Therefore, the present study investigated the ratios of 1660/cm and 1690/cm Raman shifts underlying the amide I band to indicate the cross linking of Pyr and DHLNL. Results indicated that the collagen cross-linking appears not to be the main reason for the mechanical deterioration of the disused bone.

Taken together, the present results proposed several hypothesis and mechanisms for the alterations of the disused bone to resist mechanical loading at the nanoscale. Firstly, the mineral removal from bone during disuse might lead to the weakness of the collagen-mineral and collagen–collagen interactions. The impaired collagen-mineral and collagen–collagen interactions was not be observed directly from the morphology of the MCFs without mechanical loading, but greatly contribute to the reorganization of the MCFs under loading. Secondly, the MCFs were stretched and disorganized as the first response under mechanical loading, while the MCFs became mechanically stable and provided suitable resistance against mechanical loading (Figure 4). From this point of view, at the nanoscale, a certain amount of MCFs in cortical bone offer the required mechanical properties at the price of the disorganization of the other MCFs. It should be noted that this is the response of the MCFs while bone is still experiencing elastic deformation. In future study, it is worth studying how the MCFs respond while the cortical bone is under plastic deformation or experiencing bone fracture.

The mechanical properties of bone are highly dependent on the cortical compartment of the bone. To date, there remains limited understanding on the *in situ* responses of the entire cortical bone to mechanical loading, particularly at the nanoscale. Although the AFM approach adopted in the present study offers the feasibility of revealing bone morphological alterations and deformation mechanism, it is confined to the exposed superficial layer of the MCFs. The response of the deeper MCFs in the cortical bone may also significantly contribute to the mechanical behavior of bone. Novel techniques that are based on experiments or simulations are expected to focus on this issue in future studies.

## Conclusion

In conclusion, the present study indicates that disuse induced by hindlimb unloading did not alter the orientation and D-periodic spacing of the collagen fibril in the antero-medical aspect of the cortical tibia, but results in decreased collagen crosslinking which correlates with decreased elasticity and increased susceptibility to mechanical damage. In particular, the collagen fibrils in the disused tibia were misaligned under mechanical loading. These findings presented unique adaptation regimes of the collagen fibrils in cortical bone during disuse. The observation offers the possibilities of revealing the deformation mechanisms of bone in the relevant pathological process.

## Ethics Statement

This study was carried out in accordance with the recommendations of Laboratory Animal Management Regulations, Decree No. 2 of the State Science and Technology Commission of China. The protocol was approved by the Animal Ethics and Welfare Committee of the Northwestern Polytechnical University.

## Author Contributions

PFY, XTN, HYX, JR, and PS conceived the study. PFY, XTN, ZW, LAQ, and LR collected the data. PFY, XTN, ZW, LAQ, and LR prepared the images and carried out the data analysis. PFY and PS were involved in funding acquisition. PFY, XTN, ZW, and LAQ investigated the study. PFY and PS carried out the project administration. HYX, JR, and PS supervised the study.

## Conflict of Interest Statement

The authors declare that the research was conducted in the absence of any commercial or financial relationships that could be construed as a potential conflict of interest.

## Abbreviations

AFM: atomic force microscopy
BMC: bone mineral content
BMD: bone mineral density
CDF: cumulative distribution function
DHLNL: dihydroxylysinonorleucine
HAP: hydroxyapatite
K–S: Kolmogorov–Smirnov
MCFs: mineralized collage fibrils
PMMA: polymethylmethacrylate
Pyr: pyridinoline.
