# Supplementary material for: Disuse Impairs the Mechanical Competence of Bone by Regulating the Characterizations of Mineralized Collagen Fibrils in Cortical Bone
Source: Front Physiol. 2019 Jun 21;10:775. doi: 10.3389/fphys.2019.00775 (PMC6598106; doi:10.3389/fphys.2019.00775)
Supplement: Supplementary file 1 [file Table_1.docx]

Supplementary Material

# Materials and methods

Considering that the response of murine bone to disuse at the macroscale level has been well studies in numerous previous studies, the present observations was therefore demonstrated in these supplementary materials.

## Quantification of bone surface strain under mechanical loading

Digital Image Correlation (DIC) approach was adopted to quantify the surface strain of tibia from both the control and tail suspension groups (3 right tibias of 3 mice from each group). In detail, mice tibia was demineralized according to the procedure for AFM scanning. The flat surface in the antero-medial aspect of the tibia was coated with a thin layer of white and black oil paint (Mr. Hobby C211, Gunze Sangyo, Japan).A CCD camera (50 mm lenses with the 25 mm distance ring, Daheng MER107014U3, China) mounted on a tripod was positioned in front of the loading device to provide a 20 mm × 15 mm field of view (3840 × 2748 pixels), with the depth of focus field of 6 mm. The camera system was calibrated prior to the measurements. The average pattern density was approximately 70 dots/mm^2^. Bone was loaded with 6 N in the axial tibia loading model. Images of the non-loaded bone surface were recorded as baseline to calculate the bone strain under 6 N. All images were collected at 0.5 Hz and processed using a custom-written de-noising DIC routine. Principal strain on the bone surface was computed.

## Bone mineral content, density, and structure assessments

The bone mineral density (BMD) and bone mineral content (BMC) of the hind limb bones (12 bones from all 12 mice from each group) were quantified using dual-energy X-ray absorptiometry (DEXA) *in vivo* (InAlyzer, Medikors, Seungnam, Korea). On days 0 and 28, all mice were anesthetized using intraperitoneal injection of 3% pentobarbital sodium (1.5 ml/kg) and placed in the prone position with the lower limbs naturally extended. The BMD and BMC of bilateral femur and tibia were analyzed.

The microstructure of the left murine tibia from three groups (6 tibias of 6 different mice) were quantified by a microCT scan. Bone samples were fixed with paraformaldehyde and stored until the tests. The mineral content and distribution of the bone samples were assessed using a quantitative microCT system (GE-LSP microCT, GE, Madison, WI, USA). Diaphyseal scans of the proximal tibia were taken with an 8-µm isotropic voxel resolution, with the X-ray tube operated at 80 kV and 80 µA, and a 3000-ms exposure time. Bone section with a thickness of 1.5 mm, located 1.5 mm below the growth plate, was selected as the region of interest for the 3D reconstruction and the quantitative analyses on both the trabecular and cortical bone. The chosen region of interest for cortical and trabecular bone analyses was very close to the AFM scanning locations in order to relate these data more closely. The bone surface/volume ratio (BS/BV), bone volume fraction (BV/TV), trabecular space (Tb. Sp), trabecular thickness (Tb. Th), trabecular number (Tr. N), and structure model index (SMI) were calculated. Moreover, the properties of the cortical bone, *i.e.*, BMC, cortical area, marrow area, inner perimeter, outer perimeter, and mean cortical thickness were also analyzed.

## Macro-scale Mechanical Property of Tibia Samples

The macro-scale mechanical properties of the murine tibia from all groups (6 tibias of 6 different mice from each group) were assessed using a conventional three-point bending test in a universal testing machine (Instron, USA). The support span was set at 10 mm. The orientation of the tested bone samples on the support roller were adjusted to make them visually identical. The loading speed was set at 1.5 µm/s until bone fracture. The strain-stress curve was derived from the load-deformation curve. The dimension of the cross-section area of bone fracture was measured using a caliper and stereo microscope to calculate the moment of inertia of bone about the neutral axis. The elastic modulus, stiffness, ultimate load, ultimate displacement, ultimate stress, ultimate strain, fracture load, fracture displacement, fracture stress, fracture strain, yield stress, yield strain, yield stress, overall toughness, and post-yield toughness of the tibia were calculated.

# Results

## Body mass

The body mass of the mice from the control and tail suspension groups remain at the same level prior to day 7. After day 14, the body mass of the mice in the tail suspension group was lower than that of the control (*p* < 0.05, Suppl.Table 1).

## Bone surface strain under 6 N

A typical strain distribution of bone surface under 6 N was demonstrated (Suppl.Fig. 1). Mechanical loading of 6 N resulted 1960 ± 240 με bone strain for control group and 2498 ± 310 με bone strain for the tail suspension group. Bone strain of the tail suspension group was 27% larger than that of the control group (*p* = 0.08).

## Bone mineral density of tibia

Significant differences were found on the overall BMD and BMC of murine tibia after 28 days (*p* < 0.001). The BMD and BMC of tibia in tail suspension were lower than the control (Suppl.Table 2).

## Microstructure of Bone: MicroCT Analyses

Statistical analyses identified significant differences between the control and the tail suspension groups on BV/TV. BSA/BV, Tb. Th, Tb. Th, Tr. N, Tb. Sp, Marrow Area, CSMI, SMI, Cortical Area, and Cortical BMC after 28 days (Suppl.Table 3).

## Mechanical Properties of Tibia

The elastic modulus of the murine tibia remained nearly constant after 28 days. Tail suspension disuse lead to a reduction of the fracture load and ultimate load of the tibia by 30% (p < 0.05) and 19% (p < 0.05), but slightly increased the post-yield displacement and post-yield toughness of the tibia (p < 0.05). The stiffness and the toughness of the tibia remained unchanged after 28 days.

# Supplementary Figures and Tables

## Supplementary Figure


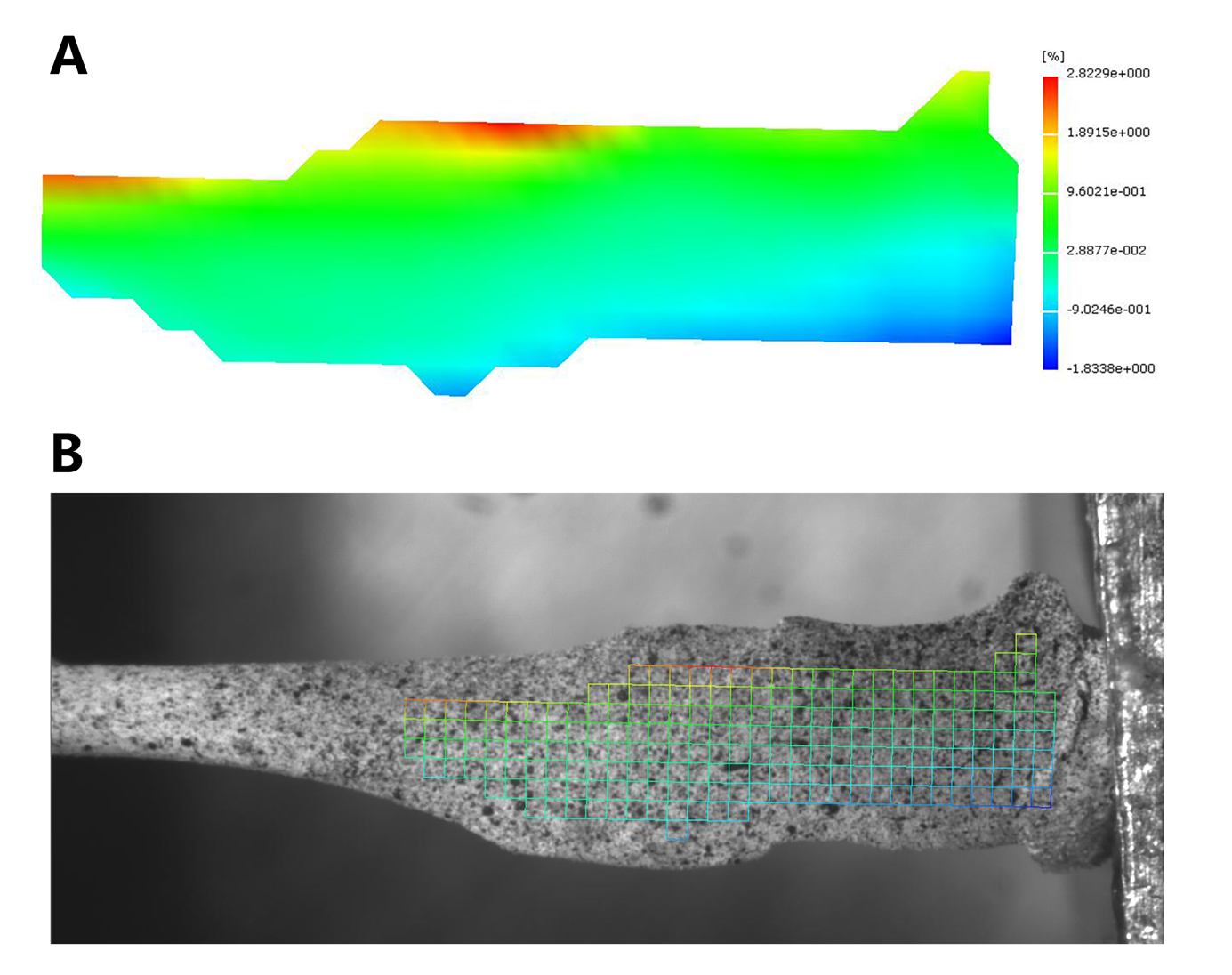


Supplementary Figure 1. Typical strain distribution of tibia surface under 6 N. The corresponding sites for AFM scanning on the bone surface was chosen for further analysis. A: bone strain distribution of tibia under 6 N, B: photo of well-prepared tibia surface for DIC assessment.

## Supplementary Tables

Suppl.Table 1. Body mass of the mice in different groups (n = 12)

| Day | Control | Tail Suspension |
| --- | --- | --- |
| 0 | 22.2 ± 1.1 | 22.0 ± 1.3 |
| 7 | 22.5 ± 1.4 | 21.8 ± 1.3 |
| 14 | 22.7 ± 1.2 | 21.2 ± 1.7* |
| 21 | 23.1 ± 1.4 | 21.7 ± 1.4* |
| 28 | 23.5 ± 1.6 | 21.9 ± 1.5* |

Data was generated from 12 bone samples of all 12 mice in each group.

Suppl.Table 2. Bone mineral density (BMD) and bone mineral content (BMC) of the tibia in mice (n = 12)

|  |  | Day |  |
| --- | --- | --- | --- |
|  |  | 0 | 28 |
| BMD (g/mm^2^) | Control | 0.092 ± 0.0063 | 0.097 ± 0.0056 |
|  | Tail Suspension | 0.094 ± 0.0065 | 0.081 ± 0.0059*** |
| BMC (g) | Control | 0.021 ± 0.0026 | 0.025 ± 0.0016 |
|  | Tail Suspension | 0.021 ± 0.0018 | 0.020 ± 0.0014*** |

Data was generated from 12 bone samples of all 12 mice in each group.

Suppl.Table 3. Microstructure of the proximal tibia in mice from different groups (n = 6)

| Variable/Group | Control | Tail Suspension |
| --- | --- | --- |
| BV/TV (%) | 12 ± 1.8 | 4.7 ± 1.6*** |
| BSA/BV (%) | 60 ± 2.2 | 82 ± 4.6*** |
| Tb. Th (mm) | 0.034 ± 0.0012 | 0.025 ± 0.0014*** |
| Tr. N | 3.6 ± 0.42 | 1.9 ± 0.58*** |
| Tb. Sp (mm) | 0.25 ± 0.032 | 0.55 ± 0.18** |
| Marrow Area (mm^2^) | 0.33 ± 0.024 | 0.37 ± 0.036 |
| CSMI (mm^4^) | 30 ± 14 | 16 ± 2.3* |
| SMI | 2.3 ± 0.13 | 3.1 ± 0.34*** |
| Cortical Area (mm^2^) | 0.60 ± 0.041 | 0.52 ± 0.031** |
| Cortical BMC (g) | 0.012 ± 0.0014 | 0.0094 ± 0.0010** |

BV/TV: Bone volume fraction, BSA/BV: Bone surface area to bone volume ratio, Tb. Th: Trabecular thickness, CWT: Cortical wall thickness, Tr. N: Trabecular number, Tb. Sp: Trabecular space. CSMI: Cross-sectional moment of inertia, SMI: Structural model index. *: Comparison with the control group. Data was generated from 6 bone samples of 6 different mice in each group. *: *p* < 0.05, **: *p* < 0.01, ***: *p* < 0.001.

Suppl.Table 4. Macro-mechanical properties of the tibia from different groups (n = 6)

| Variable/Group | Control | Tail Suspension |
| --- | --- | --- |
| Elastic Modulus (GPa) | 16 ± 1.1 | 14 ± 3.5 |
| Stiffness (N/mm) | 36.4 ± 5.3 | 31.2 ± 5.0 |
| Fracture Load (N) | 11 ± 1.7 | 7.8 ± 2.2* |
| Ultimate Load (N) | 11 ± 1.7 | 8.9 ± 0.29* |
| Toughness (J/mm^2^) | 2.7 ± 0.2 | 2.9 ± 1.0 |
| Post Yield Displacement (mm) | 0.08 ± 0.04 | 0.20 ± 0.13* |
| Post Yield Toughness (J/m^2^) | 1.2 ± 0.6 | 1.6 ± 1.0* |

Data was generated from 6 bone samples of 6 different mice in each group. *: compared with the control group. *: *p* < 0.05.
